# Classifying Diabetic and Healthy *β*-Cells in Type 2 Diabetes Using Machine Learning on Single-Cell RNA Sequencing Data

**DOI:** 10.34133/csbj.0114

**Published:** 2026-07-24

**Authors:** Daniel F. O. Onah, María De La Luz Lomboy Toledo

**Affiliations:** Department of Information Studies, University College London, London, UK.

## Abstract

Diabetes is a chronic metabolic disorder characterized by elevated blood glucose levels due to impaired insulin production or function. Two main forms are recognized: type 1 diabetes, which involves the autoimmune destruction of insulin-producing *β*-cells, and type 2 diabetes (T2D), which arises from insulin resistance and progressive *β*-cell dysfunction. Understanding the molecular mechanisms underlying these diseases is essential for the development of improved therapeutic strategies, particularly those targeting *β*-cell dysfunction. To investigate these mechanisms in a controlled and biologically interpretable setting, mouse models have played a central role in diabetes research. Owing to their genetic and physiological similarity to humans, together with the ability to precisely manipulate their genome, mice enable detailed investigation of disease progression and gene function. In particular, mouse models have provided critical insights into *β*-cell development, cellular heterogeneity, and functional failure under diabetic conditions. Building on these experimental advances, this study applies machine learning methods to single-cell transcriptomic data from mouse pancreatic islets. Specifically, we evaluate 2 supervised approaches identified in the literature, extra trees classifier and partial least squares discriminant analysis, to assess their ability to identify T2D-associated gene expression signatures at a single-cell resolution. Model performance is evaluated using standard classification metrics, with an emphasis on interpretability and biological relevance.

## Graphical Abstract

**Figure:**
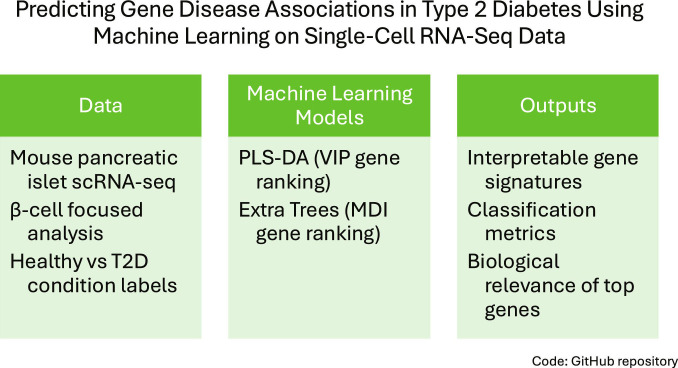

## Introduction

Advances in mouse genomics have greatly facilitated the study of type 2 diabetes (T2D) at the gene expression level. In particular, single-cell RNA sequencing (scRNA-seq) technologies enable the high-resolution profiling of individual pancreatic islet cells, capturing cell-to-cell variability that is masked in bulk analyses. Leveraging these advances, Hrovatin *et al*. [1] compiled a comprehensive cross-condition Mouse Islet Atlas (MIA), integrating over 300,000 single cells from 56 samples across 9 independent datasets. These datasets span a wide range of biological contexts, including developmental stages from embryonic to aged mice, both sexes, and multiple disease states. Importantly, the atlas incorporates several well-established diabetes models, such as the autoimmune nonobese diabetic (NOD) mouse for type 1 diabetes (T1D), the db/db model for T2D, and the *β*-cell ablation streptozotocin (STZ) model.

By combining data from healthy, immature, aged, and diseased *β*-cells, the MIA provides a unified view of how these cells change under different conditions. This approach has identified new *β*-cell states that appear during disease and revealed key molecular pathways related to stress, loss of cell identity, and compensation. In particular, *β*-cells from the db/db model, a mouse model for T2D caused by a mutation in the leptin receptor gene, which leads to obesity, insulin resistance, and progressive *β*-cell failure, showed gene expression patterns very similar to those in human T2D *β*-cells. This supports the relevance of the db/db model for studying the molecular basis of human diabetes [1].

While the MIA provides a comprehensive and unified view of *β*-cell states across diverse biological conditions, the analysis of such scRNA-seq data remains inherently challenging. Single-cell transcriptomic datasets are highly dimensional and subject to technical noise, biological variability, and batch effects, particularly when integrating data from multiple studies [2]. Although standard analytical approaches, including clustering and differential gene expression analysis, are effective for defining major cell populations and identifying marker genes, they often offer a limited representation of the complex transcriptional relationships underlying disease-associated cellular states [3]. These challenges highlight the need for integrative computational frameworks capable of systematically leveraging large-scale scRNA-seq resources to better characterize *β*-cell heterogeneity and dysfunction in T2D.

In recent years, advances in computational biology have begun to bridge this gap. Machine learning (ML) techniques provide a powerful framework for uncovering latent patterns and generating predictions from transcriptomic data. Supervised ML models can be trained to classify healthy versus diabetic cell states based on gene expression signatures, whereas unsupervised approaches can identify previously unknown cell subtypes or transitional cellular states. The availability of large, curated resources such as the MIA enables the application of these data-intensive methods with increased robustness and biological interpretability. Thus, integrating ML with scRNA-seq data holds substantial potential for advancing our understanding of *β*-cell dysfunction and accelerating the discovery of therapeutic targets in T2D.

This gap has motivated the application of ML techniques to the classification of cellular disease states from gene expression data. ML algorithms can analyze complex multivariate patterns in transcriptomic profiles and learn classifiers that distinguish diabetic from nondiabetic *β*-cells. By training on examples of known diabetic and nondiabetic cell profiles, an ML model can help identify transcriptional patterns associated with disease state and highlight candidate features for downstream biological interpretation.

Recent work by Li *et al*. [4] demonstrates the power of this approach: they analyzed scRNA-seq data from human islet cells (949 T2D and 651 control cells) using several ML algorithms and feature selection methods, including support vector machines, random forest (RF), extreme gradient boosting, and least absolute shrinkage and selection operator (LASSO)-based feature selection. This analysis discovered novel T2D-associated genes (e.g., *MTND4P24* and *MTND2P28*) and even inferred logical rules involving multiple genes that could distinguish diabetic from healthy cells. These insights were obtained by going beyond single-gene statistics to a multivariate ML framework. Such findings underscore that ML can reveal the complex gene signatures of disease that conventional analyses might miss.

### Research objectives

This study applies ML techniques to classify diabetic and nondiabetic pancreatic *β*-cells based on their gene expression signatures. By learning from high-dimensional single-cell transcriptomic data, the models aim to detect patterns associated with diabetic cellular states while retaining biological interpretability.

The study has 2 main objectives: first, to evaluate the classification performance of partial least squares discriminant analysis (PLS-DA) and extra trees classifier (ETC) on MIA *β*-cell data and, second, to identify genes that contribute most strongly to the separation between healthy and diabetic *β*-cells. Because the dataset integrates multiple studies, the analysis is presented with explicit consideration of potential batch effects, data leakage, and the limitations of internal validation.

### Methodology

First, a literature review was conducted to establish the scientific basis of the study, with emphasis on scRNA-seq analysis, pancreatic *β*-cell dysfunction in T2D, and the use of ML methods for transcriptomic classification. Next, scRNA-seq data from the MIA were filtered to retain adult pancreatic *β*-cells annotated as healthy or type 2 diabetic. The preprocessing workflow, including normalization, log-transformation, and highly variable gene selection, was implemented within the model-development pipeline to reduce the risk of data leakage. Model training and evaluation were then performed using explicitly defined data splits and cross-validation procedures. Finally, model performance was assessed using accuracy, precision, recall, *F*_1_-score, area under the receiver operating characteristic (ROC) curve (AUC–ROC), and precision–recall analysis, and gene rankings were examined for biological interpretation.

## Literature Review

### Diabetes as a disease

Diabetes mellitus is a group of metabolic disorders characterized by chronic hyperglycemia resulting from impaired insulin secretion, impaired insulin action, or both. The 2 main forms are T1D and T2D. T1D is primarily associated with autoimmune-mediated destruction of pancreatic *β*-cells, leading to an absolute deficiency of insulin, whereas T2D is characterized by insulin resistance accompanied by progressive *β*-cell dysfunction and insufficient insulin secretion [4]. As insulin demand increases, *β*-cells initially attempt to compensate, but this adaptive capacity declines over time, resulting in sustained hyperglycemia.

T2D is the predominant form of the disease, accounting for approximately 90% of all diabetes cases worldwide, and is strongly associated with obesity and environmental factors such as physical inactivity and sedentary lifestyle [4,5]. These factors impose chronic metabolic stress on pancreatic islet cells, contributing to impaired insulin sensitivity and gradual loss of *β*-cell function. Consequently, T2D is increasingly recognized as a complex and multifactorial disease involving both systemic metabolic disturbances and intrinsic alterations in pancreatic islet biology.

Diabetes has emerged as a major global public health challenge. In 2015, an estimated 415 million people were affected worldwide, and this number is projected to rise to 642 million by 2040, driven largely by the growing prevalence of T2D [5]. These trends underscore the urgent need to better understand the cellular and molecular mechanisms underlying *β*-cell dysfunction in order to support the development of more effective preventive and therapeutic strategies.

### Genetic factors and implicated genes in diabetes

T2D is a genetically complex disease in which susceptibility arises from the combined effects of numerous genetic variants, each contributing modestly to disease risk. Family and population studies indicate a strong heritable component; however, the genetic architecture of T2D is highly polygenic and shaped by interactions among multiple genes and environmental factors [6]. As a result, identifying causal genes and mechanisms has proven challenging, as many associated variants exert small effects and act within interconnected biological networks rather than isolated pathways.

Large-scale genetic studies have revealed that a substantial proportion of T2D-associated variants are linked to pancreatic islet function, particularly pathways regulating insulin secretion from *β*-cells [7]. Many risk variants are located in the noncoding regions of the genome, suggesting that altered gene regulation, rather than changes in protein sequence, plays a key role in disease development. These findings support the view that impaired *β*-cell function is a central component of T2D pathogenesis and highlight the need for integrative approaches that combine genetic variation with molecular and cellular phenotypes to better understand disease mechanisms [6,7].

In summary, T2D risk is mediated by multiple genetic variants that primarily act by subtly modulating *β*-cell function and related metabolic pathways. As genomic and analytical tools continue to advance, additional implicated genes are likely to be identified, providing promising targets for future therapeutic development and precision medicine approaches in the future.

### The MIA for diabetes analysis

Hrovatin *et al*. [1] developed the MIA, a unified single-cell transcriptomic resource that integrates more than 300,000 cells across healthy and diabetic conditions. For this study, the MIA is important because it provides a high-quality, well-annotated reference of *β*-cell states that captures how gene expression changes during diabetes. By harmonizing data from multiple diabetic models, the MIA reveals which transcriptional signatures are robust and consistently associated with *β*-cell dysfunction.

Moreover, the atlas shows that *β*-cells under metabolic or toxin-induced stress (db/db and STZ models) share strong transcriptional similarities with human T2D *β*-cells. This offers biologically relevant ground truth for distinguishing diabetic from healthy cells based solely on gene expression, which is the aim of the present analysis. The MIA also highlights key pathways altered in diabetes, such as stress-response programs and loss of *β*-cell maturity markers, providing biological context for the gene-level signals later identified by the ML models.

In summary, the MIA offers a foundational transcriptomic landscape that enables this study to explore how single-cell gene expression profiles discriminate healthy from diabetic *β*-cells and to evaluate whether ML models can capture these disease-associated patterns with high accuracy.

### ML for genomic and transcriptomic analysis

The rapid growth of high-throughput genomic technologies in diabetes research has made ML a vital tool for uncovering patterns in large and complex datasets. ML algorithms are especially useful for identifying relevant features, such as genes, single-nucleotide polymorphisms, and methylation sites, associated with disease and for building predictive models of molecular phenotypes that go beyond traditional statistical methods.

One major application is the integration of multi-omics, epigenomic, and transcriptomic information to better understand gene regulation in diabetes. Rather than analyzing each data type separately, ML methods can link them to identify shared biomarkers. For example, Rönn *et al*. applied a supervised ML method called DIABLO (Data Integration Analysis for Biomarker discovery using Latent cOmponents), based on partial least squares (PLS), to human pancreatic islet data from 110 donors, approximately 30% of whom had T2D. By integrating RNA sequencing (RNA-seq), DNA methylation, single-nucleotide polymorphism genotypes, and clinical data, the model achieved high classification performance, with an accuracy of approximately 91% and an area under the curve (AUC) of 0.96, in distinguishing T2D from control islets [7].

Importantly, the same study also evaluated a simpler ML approach using only RNA-seq data in combination with a PLS-DA model. Using a subset of 38 selected genes, this model achieved a classification accuracy of 84% to 90% and an AUC of 0.92 to 0.96, performance levels comparable to those of the full multi-omics model. These results demonstrate that even when restricted to transcriptomic data alone, supervised ML methods can identify robust gene expression signatures associated with T2D.

A deeper examination of PLS-DA illustrates why this method has become increasingly popular in genomic and transcriptomic studies. As Mehmood *et al*. emphasize, PLS-DA is particularly well suited for high-dimensional biological data, where the number of variables, such as genes, greatly exceeds the number of samples. By projecting predictors into a latent space that maximizes covariance with the response variable, PLS-DA effectively reduces dimensionality while preserving discriminative information [8,9].

The MIA provides a framework to study these phenomena by enabling direct comparison of gene expression profiles from immature, mature, and failing *β*-cells. For instance, pathways related to oxidative stress responses or fetal developmental programs may be reactivated in aging or diabetic *β*-cells, suggesting a reversion to progenitor-like features, a form of dedifferentiation observed in diabetic islets. Hrovatin *et al*. also introduced the concept of *β*-cell “compensation” versus “failure”. In a T2D context, *β*-cells initially compensate for insulin resistance by proliferating and increasing insulin secretion, but this compensatory capacity eventually fails [1]. In this setting, PLS-DA not only reduces dimensionality but also enhances the interpretability of discriminant features, making it particularly suitable for transcriptomic datasets in which correlated gene expression patterns may obscure biologically relevant signals. In genomic research, PLS-DA has been widely applied to classify disease subtypes, identify biomarker panels, and highlight molecular pathways driving group separation. Compared with traditional methods, its ability to handle multicollinearity and noise while retaining predictive accuracy positions it as a powerful supervised learning tool for uncovering disease-associated expression signatures in diabetes and related conditions [8,9].

In summary, ML provides powerful tools for analyzing genomic and transcriptomic data in diabetes research. It enables the integration of heterogeneous data types, the discovery of complex biological patterns, and the identification of candidate biomarkers that can subsequently be validated experimentally. As demonstrated by studies such as that of Rönn *et al*., ML is instrumental in revealing multilayer disease signatures, even when applied to a single data modality such as RNA-seq.

### ML for diabetes prediction

ML is widely applied in diabetes research not only to analyze genetic and transcriptomic data but also to build predictive models that support early diagnosis and risk stratification. Diabetes is a multifactorial disease influenced by genetic, clinical, demographic, and lifestyle factors. This complexity makes it well suited to ML approaches capable of handling nonlinear relationships and high-dimensional data.

ML models can integrate dozens or even hundreds of features, thereby improving prediction accuracy and enabling more nuanced insights. Commonly used algorithms include logistic regression, decision trees, ensemble methods such as RFs and gradient boosting, support vector machines, and neural networks. These models are typically trained on datasets containing variables such as age, body mass index, blood pressure, glucose levels, and family history, with the aim of predicting either current diabetic status or future disease onset.

An illustrative example is the study by De *et al*., who evaluated how gut microbiome profiles, combined with physiological and biochemical measurements, could improve the classification of T2D. Their ML models confirmed established predictors such as glycated hemoglobin (HbA1c) and fasting glucose while also identifying additional signals, including microbial abundances and lipid ratios. These findings point toward a future in which ML-based risk models integrate clinical, molecular, and microbiome data to achieve more accurate and personalized prediction [5].

Another notable example is the work by Hama Saeed, which focused on classifying T2D using only publicly available clinical datasets. The author employed several ML classifiers, including RF, decision tree, and ETC, in combination with oversampling techniques to address class imbalance. Among the models evaluated, ETC achieved the best performance, with an accuracy of 95% and an *F*_1_-score of 0.95. ETC is an ensemble learning method that constructs multiple uncorrelated decision trees using random subsets of features and samples, providing both robustness and interpretability. The model also identified fasting glucose, body mass index, age, and insulin levels as the most influential predictors for T2D, illustrating how ML can rank feature importance and highlight key risk factors [10].

ETC is particularly well suited for high-dimensional biomedical datasets due to its strong randomization strategy during tree construction. Unlike RFs, ETC builds each tree using the full training dataset and selects split thresholds at random, which reduces variance, limits overfitting, and improves computational efficiency [11]. In addition to its classification performance, ETC provides an effective embedded feature selection mechanism through impurity-based importance measures, allowing relevant variables to be ranked according to their contribution to the model.

In conclusion, ML provides powerful tools for improving diabetes prediction and diagnosis. ML models can integrate heterogeneous inputs, identify key predictors, and outperform traditional statistical approaches in many scenarios. Among candidate methods, ETC stands out as a robust, variance-reduced, and interpretable baseline for high-dimensional settings typical of genomics and transcriptomics. In multimodal contexts, combining clinical measurements with molecular or microbiome features yields additional predictive signals, as illustrated by De *et al*. and Hama Saeed. Furthermore, ensemble methods such as ETC not only enhance predictive accuracy but also provide interpretable feature rankings, enabling clinicians to better understand the factors driving model decisions. As data availability and model transparency continue to improve, ML-based prediction tools are likely to become increasingly important in personalized diabetes care.

## Methodology

**Fig. 1.**
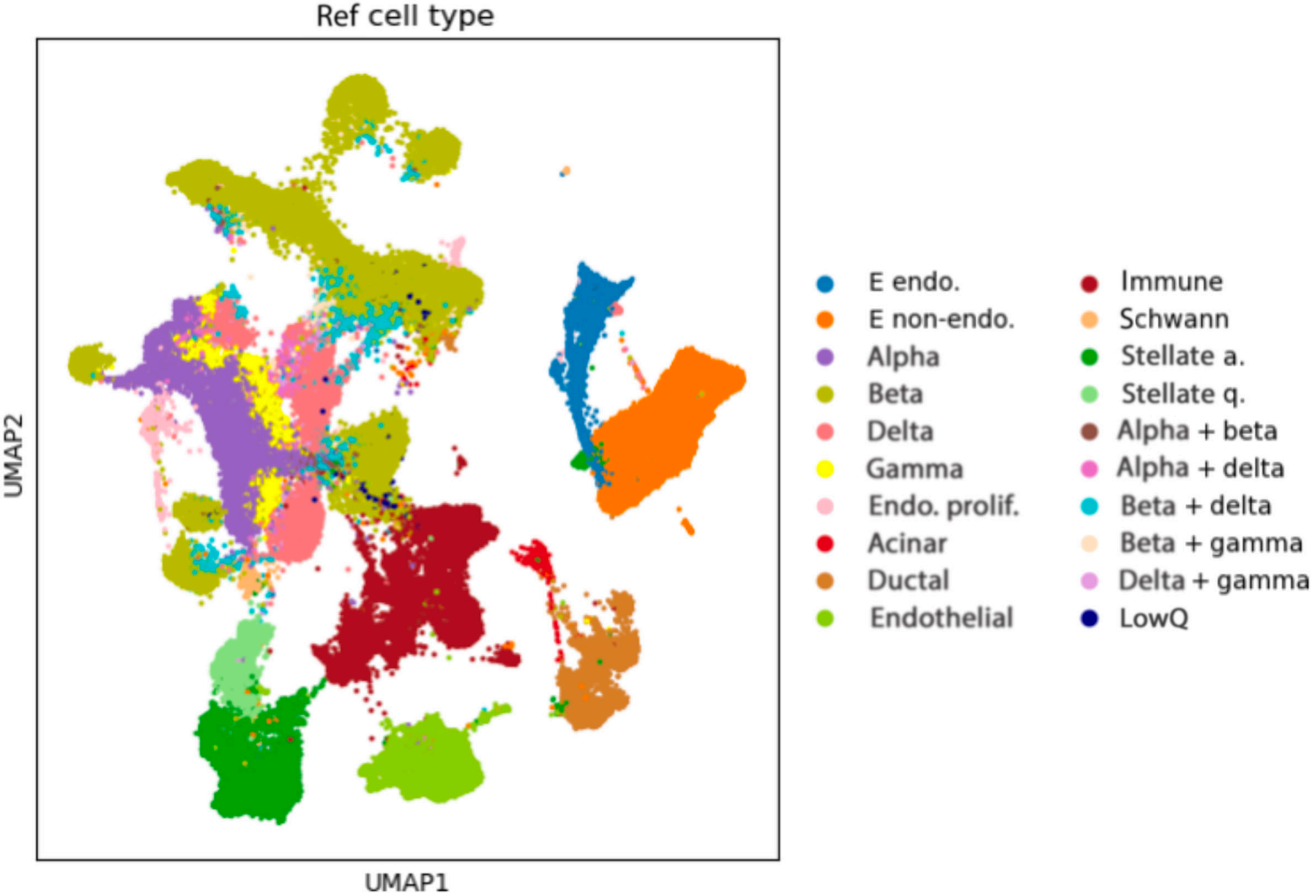
Uniform manifold approximation and projection (UMAP) visualization of annotated cell types in the Mouse Islet Atlas. Each color denotes a specific cell type, including endocrine populations (e.g., *α*, *β*, and *δ*), exocrine cells (acinar and ductal), and nonpancreatic cell types such as immune, endothelial, and stellate cells.

The methodology begins with the preprocessing and analysis of scRNA-seq data from the MIA, as shown in Fig. 1. Following an initial inspection of the available datasets, the analysis was restricted to adult pancreatic tissue by excluding the embryonic, chem, and 8-16wNOD subsets. Within the retained atlas subset, only cells annotated as normal (healthy) or type 2 diabetes mellitus (T2D) were kept for downstream analysis. To reduce confounding effects driven by differences in cell type composition, the ML preparation step was further restricted to pancreatic *β*-cells. Data filtering and handling were performed using the Python libraries Scanpy and anndata, operating on the original .h5ad files.

To reduce the risk of data leakage, the train/test split was defined before feature selection and dimensionality reduction. In this notebook, the separation was performed at the cell level, using a stratified split of the *β*-cell subset into training and held-out test partitions. This choice should be interpreted as an important limitation, because the integrated atlas may still retain a study- or sample-specific structure. The expression matrix used in the integrated atlas object had already been preprocessed upstream in the original MIA resource. As reported by Hrovatin *et al*., the downloaded data were scran-normalized, log-transformed (log(expr + 1)), and rescaled before release. This was also consistent with the values observed in the query object, which showed noninteger continuous expression values and a numerical range compatible with log-transformed data. Therefore, no additional global normalize_total or log1p transformation was applied at this stage. Instead, highly variable genes were selected on the training split only using Scanpy’s cell_ranger method with n_top_genes = 2,000. After filtering and matching the resulting feature set, the final matrix retained 1,997 highly variable genes, and the held-out test split was then restricted to the same genes without refitting. Principal component analysis (PCA) was fitted on the training matrix only, and the held-out cells were projected into the same PCA space using the fitted transformation. No batch-correction method was applied before model fitting. Because the MIA integrates multiple independent studies, any downstream classification performance should therefore be interpreted with caution and not as definitive evidence of disease-specific generalization across studies.

### Code availability

The preprocessing pipeline, ML notebooks, and analysis scripts used in this study are publicly available on GitHub (https://github.com/mdelaluzl/t2d-gene-disease-ml-prediction).

### ML models evaluated

For both models, preprocessing and feature selection steps were fit within the training data only and then applied to held-out data without refitting. Unless otherwise stated, all performance metrics reported in this study correspond to predictions on held-out data.

We tested 2 ML models that are well suited for high-dimensional biological data, PLS-DA and ETC. We begin with a description of the overall method, evaluating performance models, followed by the methodology used.

#### PLS discriminant analysis

PLS-DA is a supervised extension of PLS that constructs a small number of latent components from the high-dimensional gene matrix **X** so that these components capture as much covariance as possible with an encoded class vector or matrix **Y** (diabetic *vs.* control). In practical terms, the method learns new axes in the gene expression space along which the 2 groups differ most. Each cell is projected onto these axes to obtain scores, and classification is performed in this reduced space.

Two properties highlighted by Mehmood *et al*. [8,9] make PLS-DA attractive for scRNA-seq settings with *p* ≫ *n*:

• It handles strong multicollinearity among genes by modeling shared variation through a few supervised components.
• It yields transparent gene-level interpretation via component loadings and variable importance in projection (VIP) scores that rank features by their contribution to the discriminative components.

We adopt the VIP definition of Mahieu *et al*. [12] for PLS1:

$${\mathrm{VIP}}_{j}=\sqrt{p\frac{\sum_{h=1}^{m}R^{2}\left(y,t_{h}\right)w_{\mathrm{hj}}^{2}}{\sum_{h=1}^{m}R^{2}\left(y,t_{h}\right)}},$$ (1)

where *p* is the number of genes, *m* is the number of retained components, *R*^2^(*y*,*t_h_*) is the proportion of response variance explained by component *h*, and *w_hj_* is the loading weight of gene *j* on component *h*. For multivariate **Y** (PLS2), we follow the matrix/trace generalization described in Section 2.1 of Mahieu *et al*. [12].

In the present study, the number of retained latent components was set to 3, selected using 5-fold stratified cross-validation on the training split by maximizing the mean validation AUC over a predefined grid of 1, 2, and 3 components. This value is reported explicitly for reproducibility because the number of retained components can influence both classification performance and VIP-based gene rankings.

#### Extra trees classifier

ETC is an ensemble method that constructs a forest of highly randomized decision trees for supervised classification tasks. Unlike RFs, which rely on bootstrap sampling and search for near-optimal split thresholds within random feature subsets, ETC typically grows each tree on the full training set and introduces stronger randomization by selecting both the candidate features and their cut points at random before choosing the best split [13,14]. This additional level of randomness reduces variance and helps control overfitting, providing competitive accuracy with improved computational efficiency [13,14].

Importantly, such randomized splits are particularly advantageous in high-dimensional and noisy datasets, as they prevent the model from overfitting spurious patterns. In biomedical contexts, this property makes ETC especially well suited for omics and clinical data. For example, when applied to T2D classification tasks, ETC achieved superior AUC–ROC performance compared with other tree-based models [10]. Therefore, the method’s strong randomization not only enhances robustness against noisy biological signals but also improves interpretability through feature importance analysis.

In extra trees, feature importance is measured by the mean decrease in impurity (MDI). For a feature *j*, MDI is defined as the average reduction in node impurity whenever *j* is used for a split, weighted by the proportion of samples reaching the node, and normalized such that $\sum_{j}{\mathrm{MDI}}_{j}=1$:

$${\mathrm{MDI}}_{j}=\frac{\sum_{t=1}^{T}\sum_{n\in N_{t}}I\left\{f\left(n\right)=j\right\}\;p\left(n\right)\Delta i\left(n\right)}{\sum_{k}\sum_{t=1}^{T}\sum_{n\in N_{t}}I\left\{f\left(n\right)=k\right\}\;p\left(n\right)\Delta i\left(n\right)},$$ (2)

where Δ*i*(*n*) = *i*(*n*) − *p_L_i*(*n_L_*) − *p_R_i*(*n_R_*) and *p*(*n*) = *N_n_*/*N* denotes the proportion of samples at node *n*. Here, *i*(·) represents an impurity measure, such as the Gini index or entropy.

This criterion is fast, embedded, and widely used for variable screening. Its theoretical properties in randomized trees support its application in high-dimensional omics settings [15,16].

For ETC, the main hyperparameters explicitly set in the revised notebook were n_estimators = 1,000, max_features = sqrt, and random_state = 42 for the final held-out model. Model assessment was performed using 5-fold stratified cross-validation on the training split only, followed by final evaluation on the held-out test set. No grid search was applied in the revised notebook; therefore, the model was run using fixed settings on the training data only.

### Evaluating performance

Evaluating the performance of classification models is essential, particularly in genomics and biomedical applications where data are complex and often imbalanced. Commonly used metrics include accuracy, precision (positive predictive value), recall (sensitivity), *F*_1_-score, AUC–ROC, and precision–recall analysis. Each metric captures a different aspect of model quality, and the appropriate choice depends on the data distribution and the study objective [17]. Because the class distribution was not perfectly balanced, precision–recall analysis was also included to provide a threshold-sensitive view of model performance that is often more informative than accuracy alone under class imbalance.

In the revised workflow, the final reported metrics were derived from held-out test predictions only, whereas component selection for PLS-DA and cross-validation-based stability assessment for ETC were conducted exclusively on the training split. This separation was maintained to reduce the risk of information leakage and to ensure that performance estimates reflected evaluation on unseen data. Below, we summarize what each metric measures, its common use in genomic contexts, and key caveats such as class imbalance and other sources of bias.

#### Accuracy

Accuracy is defined as the proportion of correctly classified instances among all evaluated observations. In this study, accuracy was calculated on held-out test predictions for both PLS-DA and ETC, after all split-aware preprocessing steps had been defined from the training data. Although accuracy is easy to interpret and widely reported, it can be misleading in the presence of class imbalance because a model may achieve a high value by favoring the majority class.

For this reason, accuracy was not interpreted in isolation but together with precision, recall, *F*_1_-score, and AUC–ROC [17].

#### Precision and recall

Precision, Prec = TP/(TP + FP), quantifies the reliability of positive predictions, whereas recall (sensitivity), Rec = TP/(TP + FN), measures the ability to capture true positives. In the present study, these metrics were computed on held-out test predictions for both models. For ETC, they were also examined across 5-fold stratified cross-validation on the training split in order to assess performance stability before final evaluation on the test set.

These metrics reflect different trade-offs: increasing recall often lowers precision, resulting in more positive cases being identified at the cost of more false positives, and vice versa. Because this trade-off depends on the decision threshold, precision and recall should be interpreted jointly rather than separately, particularly in biomedical classification tasks where the relative importance of false positives and false negatives may differ [17,18].

#### *F*_1_-score

The *F*_1_-score is defined as the harmonic mean of precision and recall. It provides a single summary measure that is high only when both precision and recall are high. In this study, the *F*_1_-score was used to summarize the balance between missed diabetic cells and incorrect positive calls in the held-out test set and, for ETC, also across training-fold validation results.

#### Area under the ROC curve

AUC–ROC summarizes model performance across classification thresholds by integrating the trade-off between the true positive rate and the false positive rate [17,18]. In the revised workflow, AUC–ROC was used in 2 related ways: for PLS-DA, mean validation AUC on the training split was used to select the number of retained latent components, and the final AUC–ROC was then reported on the held-out test set; for ETC, ROC analysis was examined across 5-fold stratified cross-validation on the training split and then reported again for the held-out test evaluation.

AUC–ROC is convenient for comparing discriminative performance because it is threshold independent. However, it does not reflect the relative costs of false positives and false negatives and may appear optimistic when considered alone. For this reason, AUC–ROC was interpreted together with threshold-specific metrics, namely, precision, recall, and *F*_1_-score, at the final operating point used for classification [17].

## Findings

### Findings from PLS-DA: Classification of diabetic and healthy *β*-cell profiles from gene expression data

To examine transcriptomic differences between healthy and diabetic *β*-cells, we applied PLS-DA to the split-aware expression matrices generated in the revised preprocessing workflow. In this setting, each observation corresponds to a single *β*-cell and each predictor corresponds to one of the selected highly variable genes retained after training-based feature selection. PLS-DA projects the high-dimensional expression matrix into a small number of supervised latent components that maximize covariance with the class labels, thereby enabling classification in a reduced feature space. Because the train/test split was defined before downstream feature selection and dimensionality reduction, the results reported below correspond to held-out predictions obtained after fitting the model on the training split and applying it to the test split without refitting. Nevertheless, because the split was performed at the cell level within an integrated multistudy atlas, the findings should be interpreted with appropriate caution regarding possible batch-related structure.

#### Model training and prediction

The revised PLS-DA workflow used predefined training and held-out test objects derived from the *β*-cell subset of the MIA. After restricting the analysis to healthy and T2D labels, the training matrix contained 52,465 cells and 1,997 genes, whereas the held-out test matrix contained 13,117 cells and the same 1,997 genes. To ensure that model selection was performed on training data only, the number of retained latent components was chosen by 5-fold stratified cross-validation on the training split, using the mean validation AUC across a predefined grid of 1, 2, and 3 components. The best-performing setting retained 3 latent components. After component selection, the final model was fitted on the standardized training matrix and then applied to the held-out test matrix using the same fitted transformation.

#### Classification performance

**Fig. 2.**
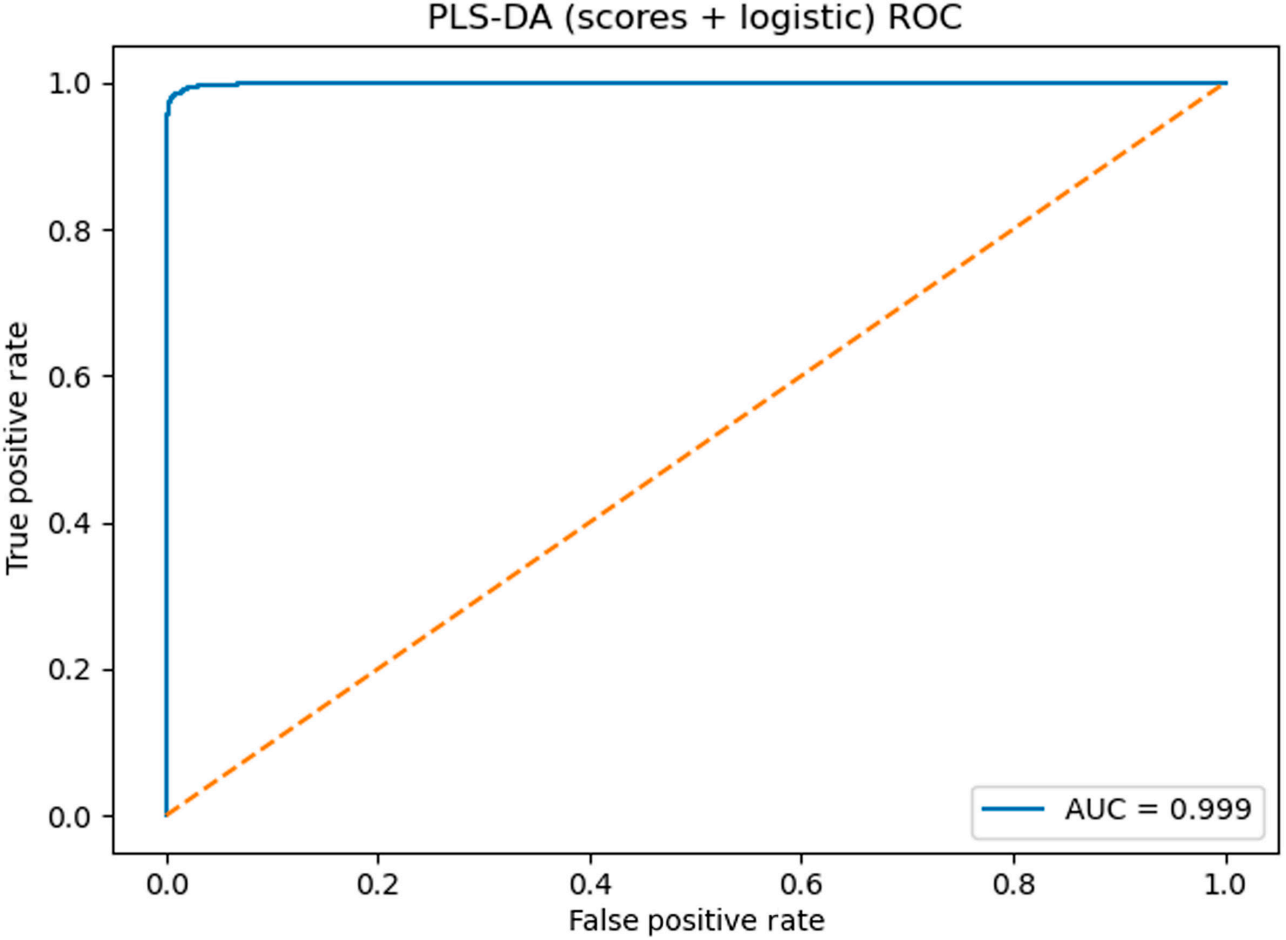
Receiver operating characteristic (ROC) curve for the partial least squares discriminant analysis (PLS-DA) model evaluated on the held-out test set. The held-out set contained 13,117 *β*-cells, and the model achieved an AUC of 0.999 after selecting the number of latent components by 5-fold stratified cross-validation on the training split.

On the held-out test set, the PLS-DA model achieved an accuracy of 98.8%, a precision of 98.7%, a recall of 98.3%, an *F*_1_-score of 98.5%, and an AUC–ROC of 0.999 (Fig. 2). These results indicate very strong separation between healthy and T2D *β*-cells under the evaluated setting. However, this performance should not be interpreted as definitive evidence of disease-specific generalization across studies, because the MIA integrates multiple datasets and no external validation or leave-one-study-out evaluation was performed in the present study. Accordingly, the ROC result is reported here as a measure of discrimination within the current held-out split rather than as proof of broad biological generalization.

#### Understanding misclassifications

**Fig. 3.**
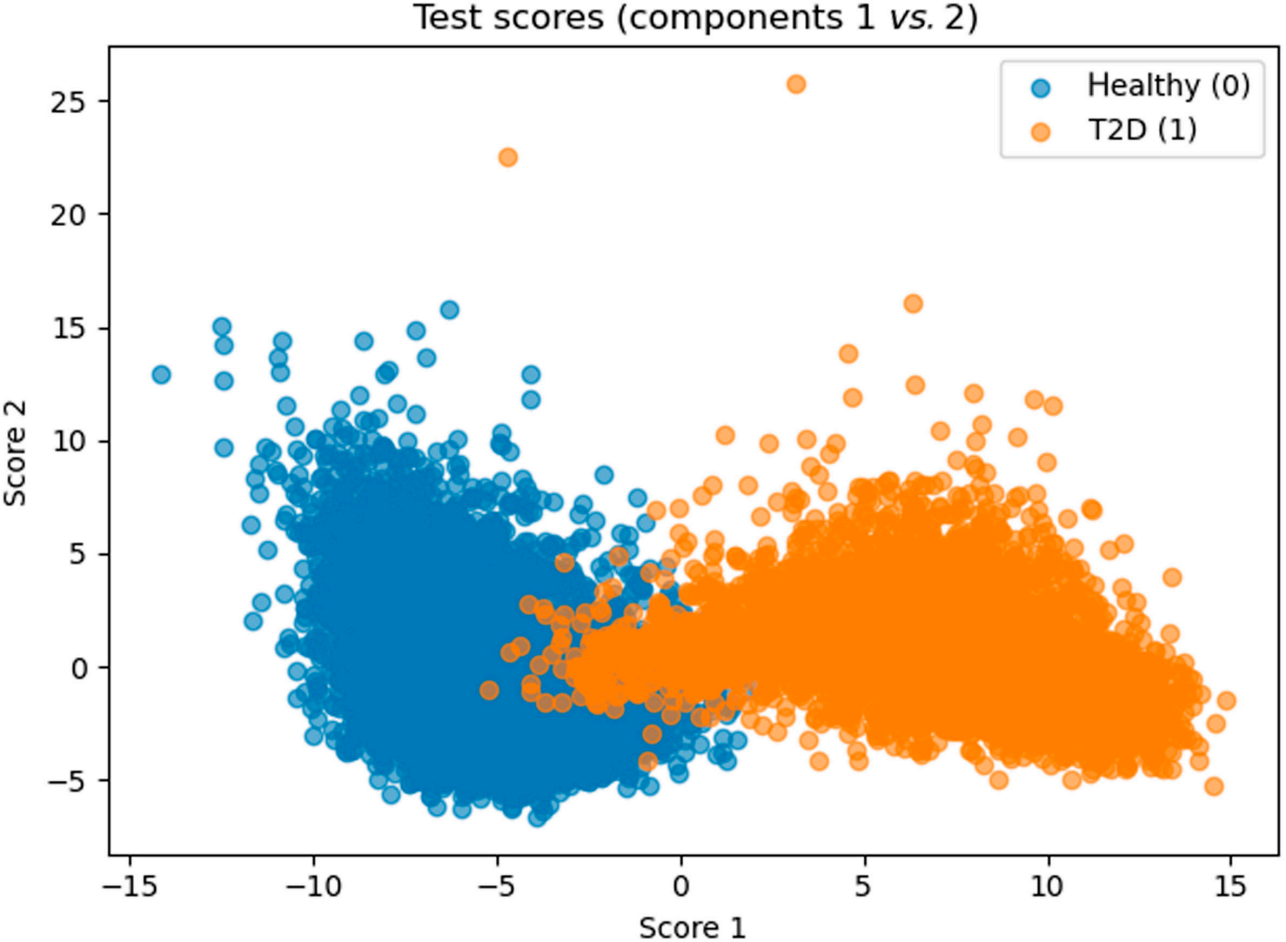
Confusion matrix summarizing the partial least squares discriminant analysis (PLS-DA) predictions on the held-out test set. Rows correspond to true labels, and columns correspond to predicted labels. Correct classifications lie along the diagonal.

The confusion matrix (Fig. 3) shows that out of 13,523 held-out test cells, 164 were misclassified. Specifically, 70 healthy cells were incorrectly labeled as T2D, whereas 94 T2D cells were incorrectly labeled as healthy. This corresponds to a confusion matrix with 7,833 true negatives, 70 false positives, 94 false negatives, and 5,526 true positives. Overall, these results indicate that the model performs strongly on the held-out split, although a small overlap remains between the 2 classes. Rather than treating the low error count as conclusive evidence of perfect biological separation, it is more appropriate to interpret these misclassifications as cells located near the decision boundary in the learned latent space.

#### Latent space representation

**Fig. 4.**
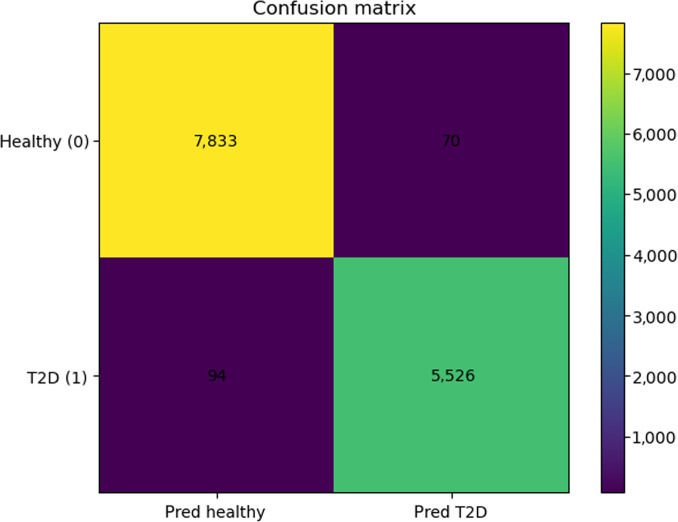
Partial least squares discriminant analysis (PLS-DA) scores for held-out test *β*-cells projected onto the first 2 latent components. Blue points correspond to healthy cells, and orange points correspond to type 2 diabetes (T2D) cells. The model retained 3 latent components in total, selected by 5-fold stratified cross-validation on the training split.

The learned separation is further illustrated in the score plot of the first 2 PLS-DA latent components (Fig. 4). Each point represents a held-out *β*-cell projected into the supervised latent space defined by the training data. Healthy cells (blue) and T2D cells (orange) occupy largely distinct regions, indicating that the fitted components capture strong class-related variation in the evaluated dataset. However, because integrated scRNA-seq datasets may retain technical structure from different studies, this visualization should be interpreted as evidence of strong separation in the present split rather than as direct proof that the latent structure is driven exclusively by disease biology.

#### Gene importance and biological interpretation

To identify which genes contributed most strongly to the separation between healthy and T2D *β*-cells, the PLS-DA model produced a ranking based on VIP scores. In practical terms, this ranking indicates which genes contributed most strongly to the discriminative latent components fitted on the training data. The highest-ranked genes are reported in Appendix A.

Several top-ranked genes are plausibly related to *β*-cell biology, including genes involved in secretory granule function, endocrine signaling, and cellular stress responses. However, these interpretations should be considered preliminary. In particular, the prominence of Gc (vitamin D-binding protein) was treated cautiously in the present study because this gene may also reflect technical or ambient RNA effects in droplet-based scRNA-seq data. Therefore, the gene rankings reported here are presented primarily as hypothesis-generating results rather than definitive disease biomarkers.

#### Conclusion

Overall, the revised PLS-DA shows that healthy and T2D *β*-cells can be separated with strong performance in the evaluated held-out split, using a workflow in which component selection was performed on training data only and the final model was applied to held-out test data without refitting. The model also provides an interpretable ranking of genes through VIP scores, which may be useful for downstream biological investigation. At the same time, the results should be interpreted within the limits of the present study: the train/test split was performed at the cell level, no explicit batch-correction method was applied before modeling, and no independent external dataset was used for validation. Therefore, the present findings support strong internal discrimination within the processed MIA subset, but they should not yet be taken as definitive evidence of robust cross-study or cross-dataset generalization.

### Findings from ETC

To complement the PLS-DA, we applied an ETC to the same split-aware *β*-cell expression matrices generated in the revised preprocessing workflow. In this setting, each observation corresponds to a single *β*-cell and each predictor corresponds to one of the selected highly variable genes retained after training-based feature selection. ETC is an ensemble classifier that builds multiple randomized decision trees and aggregates their predictions, making it suitable for high-dimensional biological data. As with the revised PLS-DA workflow, the results reported below are based on predefined training and held-out test objects, so that downstream model fitting and evaluation were carried out after split-aware preprocessing. However, because the split was performed at the cell level within an integrated multistudy atlas and no explicit batch-correction method was applied before modeling, the results should still be interpreted cautiously with respect to possible batch-related structure.

#### What was measured and how

The revised ETC workflow used predefined training and held-out test objects derived from the *β*-cell subset of the MIA. After restricting the analysis to healthy and T2D labels, the training matrix contained 54,090 cells and 1,998 genes, whereas the held-out test matrix contained 13,523 cells and the same 1,998 genes. The main hyperparameters explicitly set in the revised notebook were n_estimators = 1,000, max_features = sqrt, and random_state = 42 for the final held-out model. Other hyperparameters were left at their scikit-learn default values. Model assessment was first performed using 5-fold stratified cross-validation on the training split only, and the final model was then fitted on the full training matrix and evaluated on the held-out test matrix. Feature importance was quantified using MDI, and the highest-ranked genes are reported in Appendix B.

#### Discrimination

**Fig. 5.**
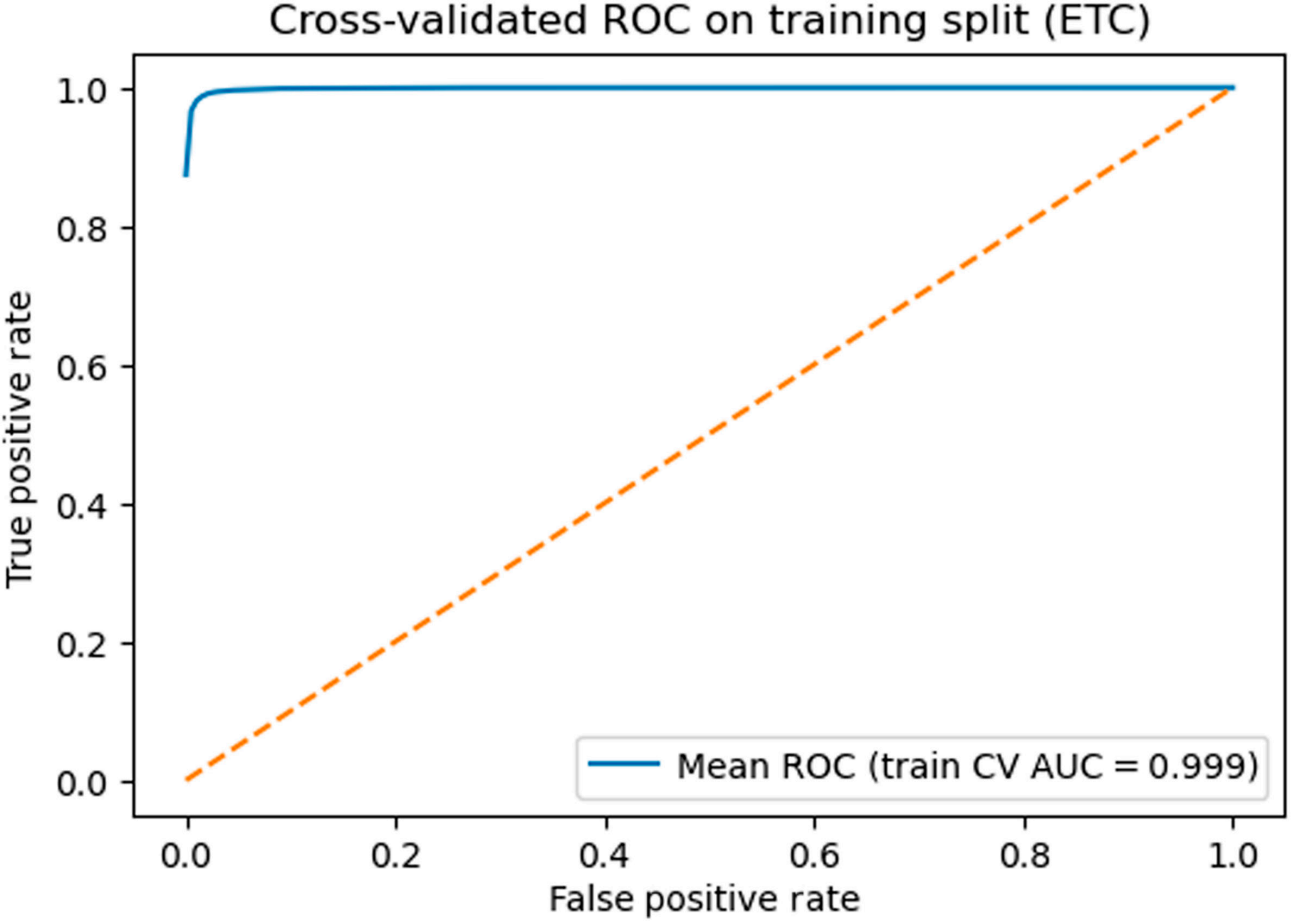
Cross-validated receiver operating characteristic (ROC) (extra trees classifier [ETC]). The curve lies near the top left corner; the mean AUC is approximately 0.999, indicating strong discrimination within the evaluated training partitions.

When the final ETC model was fitted on the full training split and evaluated on the held-out test set, it achieved an accuracy of 98.2%, a precision of 99.6%, a recall of 96.1%, an *F*_1_-score of 97.8%, and an AUC–ROC of 0.999, as shown in Fig. 5. These values indicate very strong discrimination between healthy and T2D *β*-cells in the evaluated setting.

#### Error profile (confusion matrix)

**Fig. 6.**
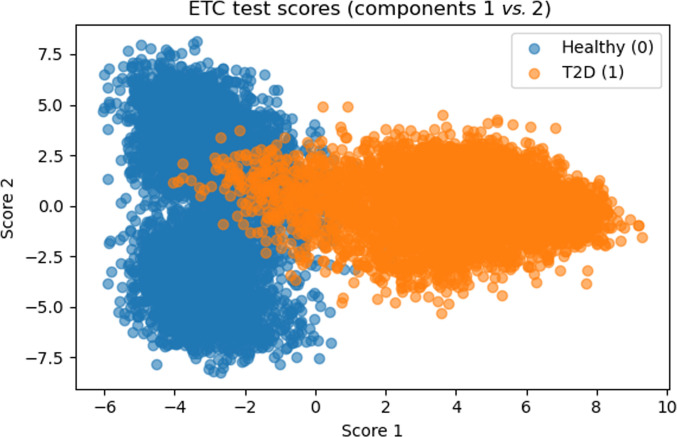
Confusion matrix for the concatenated out-of-fold predictions. Rows are true labels; columns are predicted labels.

The held-out confusion matrix shows that out of 13,523 test cells, 241 were misclassified. Specifically, 20 healthy cells were incorrectly labeled as T2D, whereas 221 T2D cells were incorrectly labeled as healthy Fig. 6. This corresponds to 7,883 true negatives, 20 false positives, 221 false negatives, and 5,399 true positives. As a result, the ETC model was highly precise in its positive calls, but its errors were not symmetric: missed T2D cells were more frequent than healthy cells incorrectly flagged as diabetic. This asymmetry is consistent with the recall being lower than the precision and should be reported explicitly, since it indicates that the model is more likely to miss some diabetic *β*-cells than to overcall healthy ones.

#### Score-space visualization

**Fig. 7.**
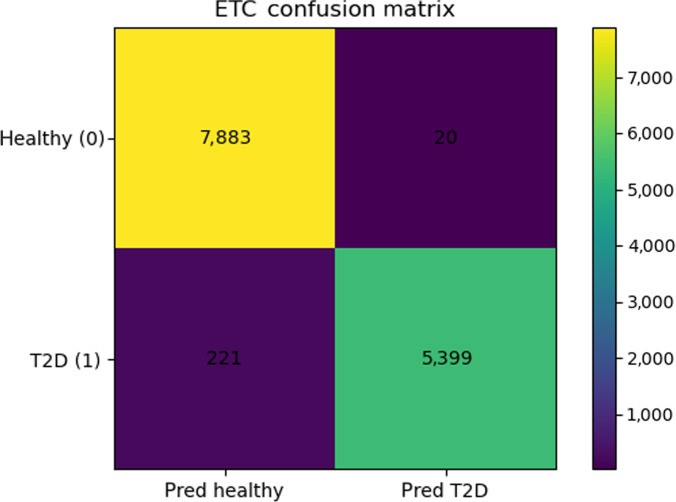
Two-dimensional principal component analysis (PCA) projection of held-out test *β*-cells used for extra trees classifier (ETC) score-space visualization. Blue points correspond to healthy cells, and orange points correspond to type 2 diabetes (T2D) cells. The PCA used for visualization was fitted on the training split only and then applied to the held-out test set.

For visualization purposes, the held-out ETC test matrix Fig. 7 was projected into a 2-dimensional PCA space fitted on the training data only. Healthy and T2D cells showed partial separation in this representation, with a limited overlap region consistent with the observed misclassifications. This plot is intended as a descriptive summary of the evaluated test split and should not be interpreted on its own as evidence of disease-specific biological generalization, particularly given the integrated multistudy nature of the dataset.

#### Gene-level signals (MDI) and biological interpretation

Appendix B reports the genes with the highest MDI scores in the final ETC model. In practical terms, these rankings indicate which genes contributed most strongly to the tree-based splits used to separate healthy from T2D *β*-cells in the evaluated dataset. Several of the top-ranked genes are plausibly related to *β*-cell biology, including genes associated with endocrine signaling, secretory function, metal ion handling, and cellular stress responses.

In the revised ETC notebook, *Gc* again appeared as the highest-ranked gene, followed by genes such as *Cck*, *Ppp1r1a*, *Fkbp11*, *Trpm5*, *Aldh1a3*, *Pam*, *Cltrn*, *Mt2*, *Cd81*, *Scg2*, and *Scg3*. While several of these genes are biologically plausible in the context of *β*-cell dysfunction, the prominence of *Gc* warrants explicit caution, because it could reflect either relevant biological signal or technical effects such as ambient RNA contamination. More broadly, the present study does not yet include pathway enrichment analysis, external validation, or stability analysis for the MDI rankings, so these gene-level findings should be interpreted primarily as candidate signals for follow-up analysis rather than definitive disease biomarkers.

At a higher level, the top-ranked ETC genes appear to relate broadly to 3 biological themes:

• insulin secretion and secretory granule function
• cellular stress and survival responses
• metabolic or signaling regulation

However, because this grouping is based on biological interpretation of the ranked genes rather than on formal enrichment testing, it should be described as a tentative summary rather than as a statistically confirmed pathway-level result.

#### Comparative interpretation (healthy *vs.* T2D)

Overall, the revised ETC analysis shows that healthy and T2D *β*-cells can be separated with strong performance in the evaluated split-aware workflow, using a model trained on predefined training data and evaluated on a held-out test set. The classifier also yields interpretable gene rankings through MDI, which may support downstream biological investigation. However, the results remain subject to important limitations: the train/test split was performed at the cell level, no explicit batch-correction method was applied before modeling, and no independent external dataset was used to validate the learned signatures. Therefore, the current ETC findings should be interpreted as evidence of strong internal discrimination within the processed MIA subset rather than as definitive proof of robust cross-study or cross-dataset generalization.

## Conclusion

This study explored the use of ML methods to classify healthy and diabetic pancreatic *β*-cells from single-cell transcriptomic data derived from the MIA. Using PLS-DA and ETC, we found that the 2 classes could be separated with strong performance in the evaluated dataset and that both models highlighted gene sets related to *β*-cell function, cellular stress, and endocrine signaling.

At the same time, the revised analysis emphasizes several important limitations. Because the MIA integrates multiple independent studies, classification performance may be influenced by technical structure in addition to disease-related biology. For this reason, the reported results should be interpreted as internal classification performance within the evaluated dataset rather than as definitive evidence of broad biological generalization. Likewise, the reported gene rankings should be regarded as candidate signals for further validation, especially for genes such as *Gc*, whose interpretation may be affected by technical artifacts in scRNA-seq data.

Overall, this study shows that interpretable ML methods can be applied to classify diabetic and healthy *β*-cells in integrated mouse scRNA-seq data while also highlighting the importance of careful preprocessing, explicit train/test separation, and cautious biological interpretation in this type of analysis. Future work should include external validation, stronger control of batch effects, and pathway-level follow-up of the identified gene signatures.

## Data Availability

The data supporting the findings of this study are available within the article and its Supplementary Materials. Additional datasets and the complete experimental code used for the analyses are available through the GitHub link provided in the article. All data and code are publicly accessible, with no restrictions on availability.

## Supplementary Materials

Supplementary 1 Appendices A and B Tables S1 and S2
