# Supplementary material for: Classifying Diabetic and Healthy β-Cells in Type 2 Diabetes Using Machine Learning on Single-Cell RNA Sequencing Data
Source: Comput Struct Biotechnol J. 2026 Jul 24;35(1):0114. doi: 10.34133/csbj.0114 (PMC13396490; doi:10.34133/csbj.0114)
Supplement: Supplementary 1 — Appendices A and B Tables S1 and S2 [file csbj.0114.f1.docx]

**Supplementary Materials**

Classifying Diabetic and Healthy *β*-Cells in Type 2

Diabetes Using Machine Learning on Single-Cell RNA-Seq Data

| 1^st^ Daniel F. O. Onah  *Department of Information Studies*  *University College London*  London, United Kingdom  d.onah@ucl.ac.uk | 2^nd^ Maria-de la Luz Lomboy Toledo  *Department of Information Studies*  *University College London* London, United Kingdom maria.toledo.24@ucl.ac.uk |
| --- | --- |

# APPENDIX A EXPLORATORY GENE RANKING BASED ON VIP SCORES FROM THE PLS-DA MODEL

Appendix A reports the top-ranked genes identified by the Partial Least Squares Discriminant Analysis (PLS-DA) model using Variable Importance in Projection (VIP) scores. VIP scores reflect how strongly each gene contributes to the latent components used for classification of healthy and T2D *β*-cells in the evaluated dataset. Accordingly, genes with higher VIP values were more influential in the model’s separation of the two classes. These rankings are presented as exploratory feature-importance results and candidate signals for follow-up analysis, rather than as definitive disease biomarkers.

# TABLE I

EXPLORATORY GENE RANKING BASED ON VIP SCORES FROM THE PLS-DA MODEL

| Gene ID | VIP | Gene name | Entrez | GO BP | GO MF | KEGG |
| --- | --- | --- | --- | --- | --- | --- |
| ENSMUSG00000035540 | 5.151889 | vitamin D binding protein | 14473 | – | – | – |
| ENSMUSG00000026335 | 4.822084 | peptidylglycine alpha-amidating monooxygenase | 18484 | – | – | – |
| ENSMUSG00000032181 | 4.728327 | secretogranin III | 20255 | – | protein binding | – |
| ENSMUSG00000022490 | 4.654675 | protein phosphatase 1, regulatory inhibitor subunit | 58200 | – | – | – |
| ENSMUSG00000003355 | 4.643532 | FK506 binding protein 11 | 66120 | – | – | – |
| ENSMUSG00000050711 | 4.617605 | secretogranin II | 20254 | – | – | – |
| ENSMUSG00000032532 | 4.532260 | cholecystokinin | 12424 | – | – | – |
| ENSMUSG00000015401 | 4.531015 | collectrin, amino acid transport regulator | 57394 | – | – | – |
| ENSMUSG00000009246 | 4.497621 | transient receptor potential cation channel, subfamily | 56843 | – | – | – |
| ENSMUSG00000018451 | 4.479327 | RIKEN cDNA 6330403K07 gene | 103712 | – | – | – |
| ENSMUSG00000031762 | 4.430531 | metallothionein 2 | 17750 | – | – | – |
| ENSMUSG00000015134 | 4.350467 | aldehyde dehydrogenase family 1, subfamily A3 | 56847 | – | – | – |
| ENSMUSG00000026989 | 4.314478 | death associated protein-like 1 | 76747 | – | – | – |
| ENSMUSG00000044139 | 4.232202 | serine protease 53 | 330657 | proteolysis | – | – |
| ENSMUSG00000031271 | 4.223421 | serine (or cysteine) peptidase inhibitor, clade | 331535 | – | – | – |
| ENSMUSG00000023236 | 4.138155 | secretogranin V | 20394 | – | – | – |
| ENSMUSG00000022315 | 4.094403 | solute carrier family 30 (zinc transporter), member | 239436 | – | – | – |
| ENSMUSG00000034871 | 4.084460 | family with sequence similarity 151, member A | 230579 | biological process | molecular function | – |
| ENSMUSG00000037706 | 4.009915 | CD81 antigen | 12520 | – | – | – |
| ENSMUSG00000045763 | 3.983353 | brain abundant, membrane attached signal protein | 70350 | – | – | – |
| ENSMUSG00000096956 | 3.927238 | small nucleolar RNA host gene 18 | 100616095 | biological process | molecular function | – |

# APPENDIX B EXPLORATORY GENE RANKING BASED ON MDI SCORES FROM THE ETC MODEL

Appendix B presents the top-ranked genes identified by the Extra Trees Classifier (ETC) based on the Mean Decrease in Impurity (MDI) measure. MDI quantifies how much each gene contributes to reducing node impurity across the ensemble of decision trees and therefore reflects its relative importance within the fitted classifier. In the present study, genes with higher MDI values contributed more strongly to the separation between healthy and T2D *β*-cells in the evaluated dataset. Please note that these rankings should be interpreted as exploratory feature-importance results rather than definitive disease biomarkers.

# TABLE II

TOP-RANKED GENES IDENTIFIED BY THE EXTRA TREES CLASSIFIER (ETC) USING MEAN DECREASE IN IMPURITY (MDI).

| Gene | Importance | Symbol | Name | Entrez | GO BP | GO MF |
| --- | --- | --- | --- | --- | --- | --- |
| Gc | 0.027922 | Gc | vitamin D binding protein | 14473 | – | – |
| Cck | 0.025458 | Cck | cholecystokinin | 12424 | – | – |
| Ppp1r1a | 0.021035 | Ppp1r1a | protein phosphatase 1, regulatory inhibitor subunit 1A | 58200 | – | – |
| Fkbp11 | 0.020510 | Fkbp11 | FK506 binding protein 11 | 66120 | – | – |
| 6330403K07Rik | 0.020364 | 6330403K07Rik | RIKEN cDNA 6330403K07 gene | 103712 | – | – |
| Trpm5 | 0.018959 | Trpm5 | transient receptor potential cation channel, subfamily M, member 5 | 56843 | – | – |
| Aldh1a3 | 0.018880 | Aldh1a3 | aldehyde dehydrogenase family 1, subfamily A3 | 56847 | – | – |
| Pam | 0.017137 | Pam | peptidylglycine alpha-amidating monooxygenase | 18484 | – | – |
| Cltrn | 0.015820 | Cltrn | collectrin, amino acid transport regulator | 57394 | – | – |
| Mt2 | 0.015611 | Mt2 | metallothionein 2 | 17750 | – | – |
| Serpina7 | 0.015476 | Serpina7 | serine (or cysteine) peptidase inhibitor, clade A, member 7 | 331535 | – | – |
| Cd81 | 0.015232 | Cd81 | CD81 antigen | 12520 | – | – |
| Prss53 | 0.014823 | Prss53 | serine protease 53 | 330657 | proteolysis | – |
| Fam151a | 0.014406 | Fam151a | family with sequence similarity 151, member A | 230579 | biological process | molecular function |
| Dapl1 | 0.013695 | Dapl1 | death associated protein-like 1 | 76747 | – | – |
| Slc30a8 | 0.012929 | Slc30a8 | solute carrier family 30 (zinc transporter), member 8 | 239436 | – | – |
| Xist | 0.012655 | Xist | inactive X specific transcripts | 213742 | – | – |
| Scg2 | 0.011922 | Scg2 | secretogranin II | 20254 | – | – |
| Scg3 | 0.011915 | Scg3 | secretogranin III | 20255 | – | protein binding |
| Basp1 | 0.011877 | Basp1 | brain abundant, membrane attached signal protein 1 | 70350 | – | – |
